# Non-synaptic Cell-Autonomous Mechanisms Underlie Neuronal Hyperactivity in a Genetic Model of *PIK3CA*-Driven Intractable Epilepsy

**DOI:** 10.3389/fnmol.2021.772847

**Published:** 2021-11-26

**Authors:** Achira Roy, Victor Z. Han, Angela M. Bard, Devin T. Wehle, Stephen E. P. Smith, Jan-Marino Ramirez, Franck Kalume, Kathleen J. Millen

**Affiliations:** ^1^Neuroscience Unit, Jawaharlal Nehru Centre for Advanced Scientific Research (JNCASR), Bengaluru, India; ^2^Center for Integrative Brain Research, Seattle Children’s Research Institute, Seattle, WA, United States; ^3^Department of Biology, University of Washington, Seattle, WA, United States; ^4^Graduate Program in Neuroscience, University of Washington, Seattle, WA, United States; ^5^Department of Pediatrics, University of Washington, Seattle, WA, United States; ^6^Department of Neurological Surgery, University of Washington, Seattle, WA, United States; ^7^Department of Physiology and Biophysics, University of Washington, Seattle, WA, United States; ^8^Department of Pharmacology, University of Washington, Seattle, WA, United States

**Keywords:** PI3K, epilepsy, mouse model, electrophysiology, hippocampus, BKM120 (buparlisib), RAD001 (everolimus), AZD5363 (PubChem CID: 25227436)

## Abstract

Patients harboring mutations in the PI3K-AKT-MTOR pathway-encoding genes often develop a spectrum of neurodevelopmental disorders including epilepsy. A significant proportion remains unresponsive to conventional anti-seizure medications. Understanding mutation-specific pathophysiology is thus critical for molecularly targeted therapies. We previously determined that mouse models expressing a patient-related activating mutation in *PIK3CA*, encoding the p110α catalytic subunit of phosphoinositide-3-kinase (PI3K), are epileptic and acutely treatable by PI3K inhibition, irrespective of dysmorphology. Here we report the physiological mechanisms underlying this dysregulated neuronal excitability. *In vivo*, we demonstrate epileptiform events in the *Pik3ca* mutant hippocampus. By *ex vivo* analyses, we show that Pik3ca-driven hyperactivation of hippocampal pyramidal neurons is mediated by changes in multiple non-synaptic, cell-intrinsic properties. Finally, we report that acute inhibition of PI3K or AKT, but not MTOR activity, suppresses the intrinsic hyperactivity of the mutant neurons. These acute mechanisms are distinct from those causing neuronal hyperactivity in other AKT-MTOR epileptic models and define parameters to facilitate the development of new molecularly rational therapeutic interventions for intractable epilepsy.

## Introduction

Mutations in the PI3K-AKT-MTOR signaling pathway, long studied for roles in cancer (Yang et al., 2019; Madsen, 2020), also cause clinically important developmental brain overgrowth syndromes. Affected individuals display phenotypes ranging from dysplastic megalencephaly, hemimegalencephaly, and focal cortical dysplasia (FCD), as well as comorbidities including hydrocephalus, autism, and intellectual disability (Bast et al., 2006; Blumcke et al., 2011; Stafstrom and Carmant, 2015; Crino, 2016; Mirzaa et al., 2018; Dobyns and Mirzaa, 2019). These mutations also cause focal epilepsy, representing 25–50% of all cases of intractable (treatment-resistant) epilepsy in children (Bast et al., 2006; Blumcke et al., 2011; Stafstrom and Carmant, 2015; Mirzaa et al., 2018; Kim and Lee, 2019; Rademacher and Eickholt, 2019; Lee et al., 2021). Most current anti-seizure drugs target single ion channels because they were developed in acute wild-type rodent seizure models (Kehne et al., 2017; Barker-Haliski and White, 2019; Wilcox et al., 2020). These models do not mimic genetic epilepsies in patients; hence the current therapeutics are largely ineffective. Since PI3K pathway mutations are now known to cause intractable epilepsy, repurposing pathway-targeted anti-cancer drugs offers a tantalizing opportunity to fundamentally shift the therapeutic approach toward intractable epilepsy. Presently, the MTOR inhibitor rapamycin and its analogs are the sole pathway-related drugs used clinically to treat epilepsy, primarily in tuberous sclerosis (TSC) patients with rare *TSC1/2* deletion mutations which enhance downstream MTOR signaling (Crino, 2016; Kim and Lee, 2019; Stafstrom, 2019). Yet, these treatments remain effective only to a modest degree, suggesting that MTOR activation may not be the sole arm of this complex signaling pathway accounting for all PI3K pathway-driven epilepsies (Cho, 2011; Meng et al., 2013; Roy et al., 2015; Nguyen and Bordey, 2021).

Mosaic activating mutations of the p110α catalytic subunit of phosphoinositide 3-kinase (*PIK3CA*) in the brain result in a spectrum of segmental overgrowth syndromes including intractable pediatric epilepsy. We have previously generated the first genetic mouse models of patient-related *PIK3CA* mutations that recapitulate brain overgrowth, cortical dysplasia, hydrocephalus, and epilepsy, with phenotypic severity dependent on the mutant allele and its time of activation (Roy et al., 2015, 2019). Moreover, this developmental epilepsy is dissociable from dysmorphology and seizures acutely suppressible by 1-h *in vivo* administration of the pan-PI3K inhibitor BKM120 (Maira et al., 2012; Roy et al., 2015). To assess the underlying acute physiological and pathway mechanisms, here we perform *in vivo* and *ex vivo* electrophysiological studies in our megalencephalic *Nestin-cre;Pik3ca^*E545K*^* mouse model, focusing on hippocampal pyramidal neurons. Hippocampal abnormalities are commonly observed in epilepsy patients (Chatzikonstantinou, 2014; Roy et al., 2020). The association between epilepsy and hippocampal pathophysiology is also well established (de Curtis and Gnatkovsky, 2009; Cho, 2011; Berdichevsky et al., 2013; Roy et al., 2015; Mazumder et al., 2019), with some evidence for hippocampal seizure onset in patients with mutations in PI3K pathway (Jansen et al., 2015). Further in this study, we demonstrate clear evidence of hippocampus-initiated seizure activity *in vivo*, which we leverage for more extensive *ex vivo* analyses. We report that *Pik3ca*^*E545K*^ gain-of-function mutation causes intrinsic changes in neuronal properties leading to hyperactivity. We further establish that this hyperactivity is acutely dependent on PI3K and AKT, but not MTOR, regulation. Our study is the first to define key aspects of acute neuronal dysregulation in this important genetic model of Pik3ca-driven epilepsy, and demonstrates these mechanisms are distinct from synaptic and network-related mechanisms reported in MTOR, TSC, and RHEB epilepsy models (Crino, 2016; Kim and Lee, 2019; Stafstrom, 2019; Hsieh et al., 2020) with PI3K pathway dysregulation.

## Results

### *In vivo* Recordings Demonstrate Network Hyperexcitability in Mutant CA1

**FIGURE 1:**
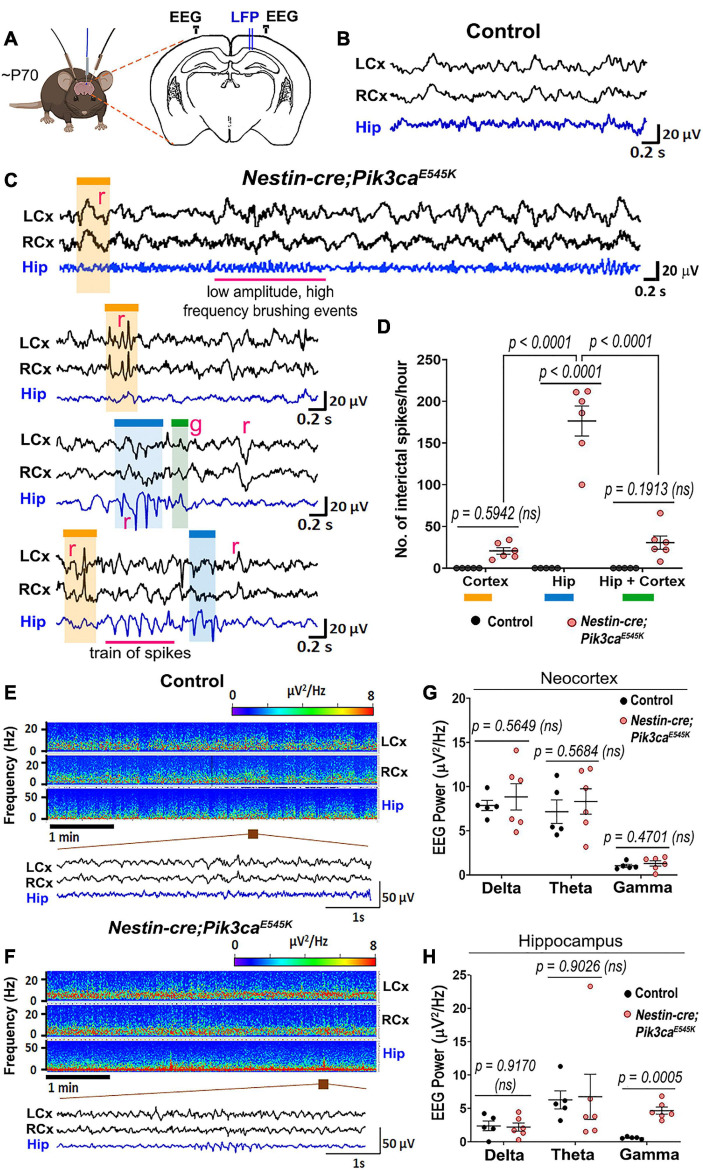
*Nestin-cre;Pik3ca^*E545K*^* mutant brains show higher neural excitability. **(A)** Schematic shows electrode placement for EEG-ECoG recordings in ∼P70 *Nestin-cre;Pik3ca^*E545K*^* and control littermates. LFP, local field potential. **(B,C)** Compared to controls, the mutants showed significantly higher frequency of regional (r) or generalized (g) spikes, train of spikes/polyspikes in neocortex (black traces) and hippocampus (blue traces). Low amplitude, high frequency brushing events were also observed in the hippocampus. **(D)** In the mutant, interictal spike frequency was significantly higher in the hippocampus (blue box), compared to those generated in neocortex (orange box) or generalized in both regions (green box) [*F* = 46.65, degrees of freedom (df) = 27]. **(E,F)** Power spectrum analysis displayed increased activity in mutants, as emphasized in the representative magnified segments. **(G,H)** Mutant hippocampus demonstrated significantly higher activity in the gamma frequency range (*F* = 3.932, df = 27; neocortex: *F* = 26.93, df = 27), where frequency bands as evaluated are: Delta: 0.5–5 Hz, Theta: 5–10 Hz, Gamma: 20–30 Hz. Data is represented as mean ± SEM scatter plots; differences were considered significant at *p* < 0.05; ns, not significant. Scale bars: 0.2 s, 20 μV **(B,C)**; 1 s, 50 μV **(E,F)**.

We investigated *in vivo* changes in the hippocampal CA1 network activity of ∼P70 *Nestin-cre;Pik3ca^*E545K*^* mutant mice relative to control littermates using local field potential (LFP) recordings, while simultaneously monitoring cortical surface activity by electrocorticography (ECoG). These recordings showed spontaneous interictal spike activity either restricted to the cortex or the hippocampus exclusively, or concurrent to the two regions in the mutant mice (Figures 1A–D). A broad spectrum of spike patterns was observed, consisting of single or groups of interictal spikes, the simplest identifiable unit of epileptiform activity (McCormick and Contreras, 2001), as well as trains of sharp spikes, low-frequency slow waves or high-frequency low-amplitude “brushing events.” These epileptiform events were observed exclusively in the mutant mice. Within the mutant group, the event frequency was significantly higher in the hippocampus than in the neocortex. Power spectrum analyses revealed that the mutant hippocampi exhibit significantly higher power in the gamma frequency bands (Figures 1E–H), which is often indicative of seizure onset (Lee et al., 2000; Hughes, 2008). There is increasing evidence in the field that elevated EEG gamma activity (20–30 Hz) can serve as a biomarker of pathological tissue involved in seizure generation or at risk of epileptogenesis (Hughes, 2008). Increased gamma activity in the hippocampus has also been associated with seizure generation in animal models (de Curtis and Gnatkovsky, 2009; Jones et al., 2015). In the same way, our data established a clear link between hippocampus and seizures in our model and demonstrated that Pik3ca overactivation caused significant neural hyperexcitation in our mice, predominantly in the hippocampus.

### Mutant CA1 and CA3 Pyramidal Neurons Are Hyperactive

**FIGURE 2:**
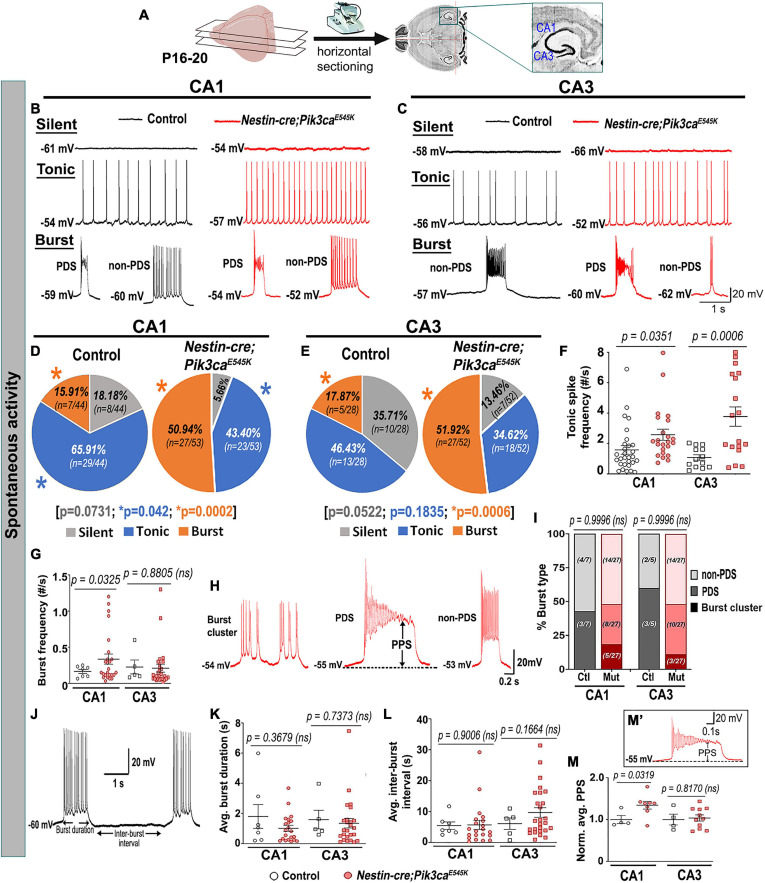
Mutant hippocampal neurons produce increased epileptiform burst activity. **(A)** Flowchart shows acute horizontal brain slicing for whole-cell recording. **(B–E)** Traces represent silent, tonic, and burst categories of CA1 and CA3 neurons based on spontaneous cellular activity; respective pie charts marked proportion of recorded cells. Mutant CA1 and CA3 exhibited significantly higher proportions of burst-firing cells compared to controls. Significantly fewer tonic-firing cells were observed in mutant CA1. **(F,G)** Relative to controls, spontaneous tonic spike frequencies were significantly higher in mutant CA1 (*t* = 2.175, df = 43.71) and CA3 (*t* = 4.063, df = 19.78) cells; burst frequency was significantly higher in mutant CA1 (CA1: *t* = 2.244, df = 29.38; CA3: *t* = 0.1561, df = 6.754). **(H)** Representative traces for subtypes of burst firing, namely burst cluster, paroxysmal depolarization shift (PDS) and non-PDS plateau bursts. **(I)** Proportion of burst subcategories were not overtly different in control and mutant CA1 and CA3 regions (*F* = 7.355e^– 12^; df = 6); burst clusters were only seen in mutant cells. **(J)** Representative trace demonstrates how burst duration and inter-burst interval were calculated. **(K,L)** In both CA1 and CA3, average (avg.) burst duration (CA1: *t* = 0.9644, df = 6.793; CA3: *t* = 0.3508, df = 6.229) and inter-burst interval (CA1: *t* = 0.1263, df = 22.31; CA3: *t* = 1.485, df = 10.73) were not significantly different between control and mutant neurons. **(M’)** Plateau potential shift (PPS) in mutant bursting cells, as depicted in panel **(M’)**, was significantly higher in CA1 (*t* = 2.586, df = 8.118) but similar in CA3 (*t* = 0.2436, df = 5.110), compared to respective controls. Data is represented as pie charts, % bar graphs and mean ± SEM scatter plots; differences were considered significant at *p* < 0.05; ns, not significant; PPS, plateau potential shift. Scale bars: 1 s, 20 mV **(B,C,J)**; 0.2 s, 20 mV **(H)**; 0.1 s, 20 mV **(M’)**.

To investigate mechanisms driving the mutant epileptiform activity at the cellular level, we used whole-cell patch-clamp recordings from CA1 and CA3 pyramidal neurons in P16–20 control and mutant brains. Silent and spontaneously firing, tonic, and burst-generating neurons were detected in both control and mutant slices (Figures 2A–C). However, mutant slices had a significantly higher proportion of burst-generating cells in both regions and lower proportion of tonic-firing cells in mutant CA1 compared to controls. Additionally, we observed significantly higher tonic spike frequencies in mutant CA1 and CA3 and higher burst frequency in mutant CA1, compared to controls (Figures 2D–G).

Burst-generating cells demonstrated multiple burst types. Specifically, we identified burst clusters and two types of plateau-bursts: paroxysmal depolarization shift (PDS) and non-PDS waveforms. We defined “burst cluster” as a multi-spike burst activity of random nature without a plateau potential; these were only observed in mutant slices (Figures 2H,I). Plateau-bursts with depolarization shift resulting in sodium-spike inactivation were termed as PDS “bursting cells.” These depolarization shifts have previously been implicated as the intracellular correlate of *in vivo* interictal spikes (McCormick and Contreras, 2001; Marcuccilli et al., 2010; Kubista et al., 2019; Tryba et al., 2019). We defined non-PDS bursts as those where plateau potential developed in absence of prominent sodium-spike inactivation. No significant differences between control and mutant hippocampal pyramidal cells were observed with respect to average burst duration or inter-burst interval. However, the plateau potential shift (PPS), defined here as the difference of the steady state plateau potential and the resting membrane potential (RMP), was significantly larger in mutant CA1 but not in CA3, relative to respective controls (Figures 2J–M,M’). This was despite similar RMP across control and mutant hippocampal neurons (Table 1).

**TABLE 1:** Summary table of intrinsic membrane properties.

**#**	**Intrinsic properties**	**CA1**		**CA3**	
		**Control**	**Mutant**	**Control**	**Mutant**
1	Resting membrane potential (RMP; mV)	−55.36 ± 0.58	−54.64 ± 0.64	−56.07 ± 0.82	−56.49 ± 0.92
2	Input resistance (MΩ)	284.27 ± 21.6	262.82 ± 22.72	331.73 ± 23.54	272.99 ± 39.14
3	Time constant (ms)	21.79 ± 1.76	23.58 ± 1.85	32.23 ± 7.33	50.78 ± 3.76
4	Rheobase (pA)	11.54 ± 3.17	18.95 ± 3.66	18.95 ± 3.32	19.33 ± 4.19

*Summary of basic intrinsic membrane properties quantitated and compared between control and mutant CA1 and CA3 neurons. No significant differences in resting membrane potential, input resistance and rheobase current (minimum injected current required to elicit the first action potential) were observed between control and mutant neurons, in both CA1 and CA3. Decay time constant of mutant CA3 neurons was significantly longer than that of control CA1 (*p* < 0.0001) and CA3 (*p* = 0.0071) cells, as well as of mutant CA1 neurons (*p* < 0.0001; *F* = 31.21, df = 33). Data is represented as mean ± SEM; differences were considered significant at *p* < 0.05.*

**FIGURE 3:**
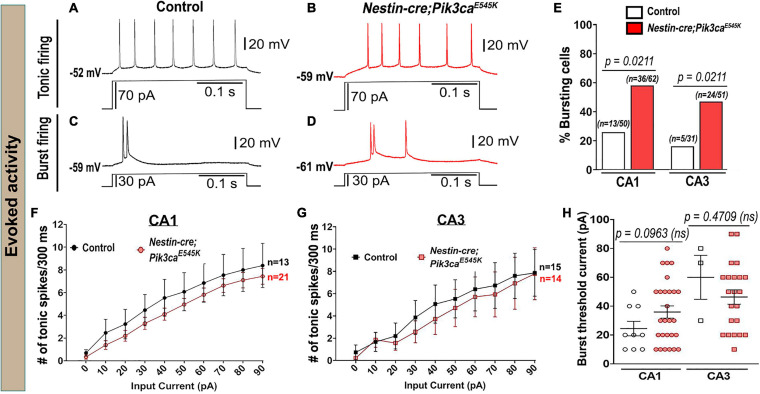
Evoked whole-cell recording shows increased bursting in mutants. **(A–D)** Representative evoked voltage traces of control and mutant showed tonic and burst firing in response to current steps. **(E)** Evoked recording also marked significantly higher bursting cell proportions in the mutant. **(F,G)** No significant differences in evoked tonic spike frequencies were observed in the tested range of input depolarizing current (0–90 pA), between control and mutant CA1 and CA3 neurons (CA1: *F* = 13.07, df = 320; CA3: *F* = 4.844, df = 270). **(H)** No overt differences in the current inducing the first burst were observed between control and mutant (CA1: *t* = 1.744, df = 20.29; CA3: *t* = 0.8493, df = 2.439). Data is represented as bar graphs, mean ± SEM scatter and line plots; differences were considered significant at *p* < 0.05; ns, not significant. Scale bars: 0.1 s, 20 mV **(A–D)**.

The evoked current-clamp recordings further validated the spontaneous activity results, especially by displaying a significantly higher percentage of burst-generating pyramidal neurons in the mutant hippocampus (Figures 3A–E). Incidentally, no significant difference in membrane intrinsic properties like input resistance, rheobase current and burst-threshold current, as well as evoked tonic spike frequencies (for the tested 0–90 pA range), was observed between control and mutant cells (Table 1 and Figures 3F–H). But the decay time constant for mutant CA3 neurons was significantly longer than that in mutant CA1 and control groups (Table 1). With similar resistance, this implied that mutant CA3 neurons have higher membrane capacitance than other cell groups. Together, these data demonstrate that Pik3ca overactivation results in intracellular neuronal hyperactivity, with some distinct cell type-specific effects.

### Pik3ca-Driven Neuronal Hyperactivity Is Not Primarily Dependent on Synaptic Inputs

**FIGURE 4:**
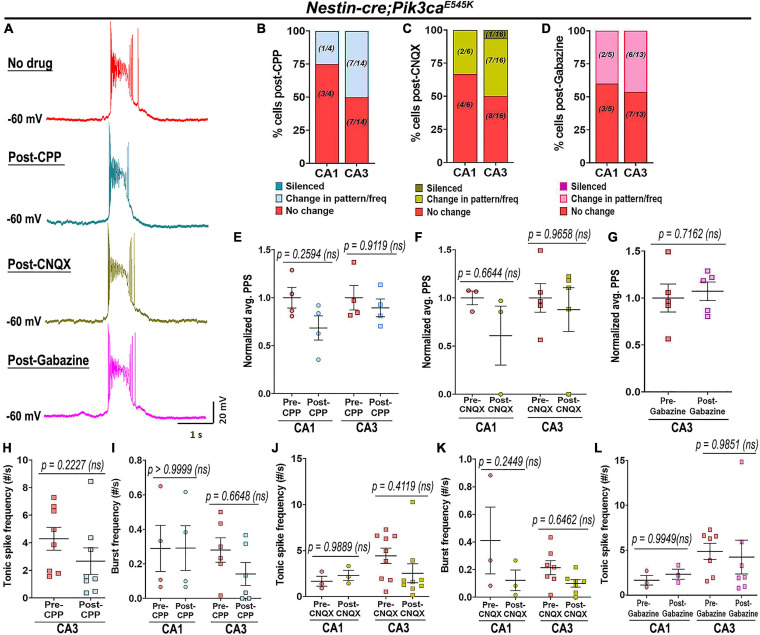
Pik3ca-related epileptiform activity is not dependent on glutamatergic or GABAergic inputs. **(A)** Representative spontaneous mutant trace and traces of the same cell post-treatment with glutamatergic receptor antagonists (CPP, CNQX) and with GABA antagonist gabazine showed no significant change in firing pattern. **(B–D)** Large proportion of mutant cells remained unaffected after being blocked from external glutamatergic or GABAergic inputs. **(E–L)** No significant differences in normalized plateau potential shift (PPS; CPP: *F* = 3.391, df = 12; CNQX: *F* = 1.432, df = 12; Gabazine: *t* = 0.3904, df = 4) or in tonic (CPP: *t* = 1.876, df = 7; CNQX: *F* = 0.2766, df = 20; Gabazine: *F* = 0.0001130, df = 16) and burst frequencies (CPP: *F* = 0.5140, df = 16; CNQX: *F* = 5.218, df = 16) were observed before and after administration of channel blockers. Data is represented as % bar graphs and mean ± SEM scatter plots; differences were considered significant at *p* < 0.05; ns, not significant. Scale bar: 1 s, 20 mV **(A)**.

To determine whether the Pik3ca-related neuronal hyperactivity is driven by altered synaptic interactions or other intrinsic properties, we assessed the effects of channel and receptor blockers on mutant hippocampal physiology. Blocking glutamatergic inputs by extracellular administration of NMDA and non-NMDA receptor-antagonists, 3-[(±)2-carboxypiperazin-4yl] propyl-1-phosphate (CPP) and 6-cyano-7-nitroquinoxaline-2,3-dione (CNQX) respectively, had no overt physiological effect on the majority of mutant hippocampal neurons (Figures 4A–C,E,F,H–K). Similarly, blocking inhibitory synaptic inputs with gabazine did not significantly alter the mutant firing patterns, spike frequencies or PPS (Figures 4A,D,G,L). The proportion of mutant CA1 neurons affected by these channel blockers was comparatively less than that in CA3. Our data lead to the important conclusion that Pik3ca-driven neuronal hyperactivity is primarily not dependent on synaptic transmission.

**FIGURE 5:**
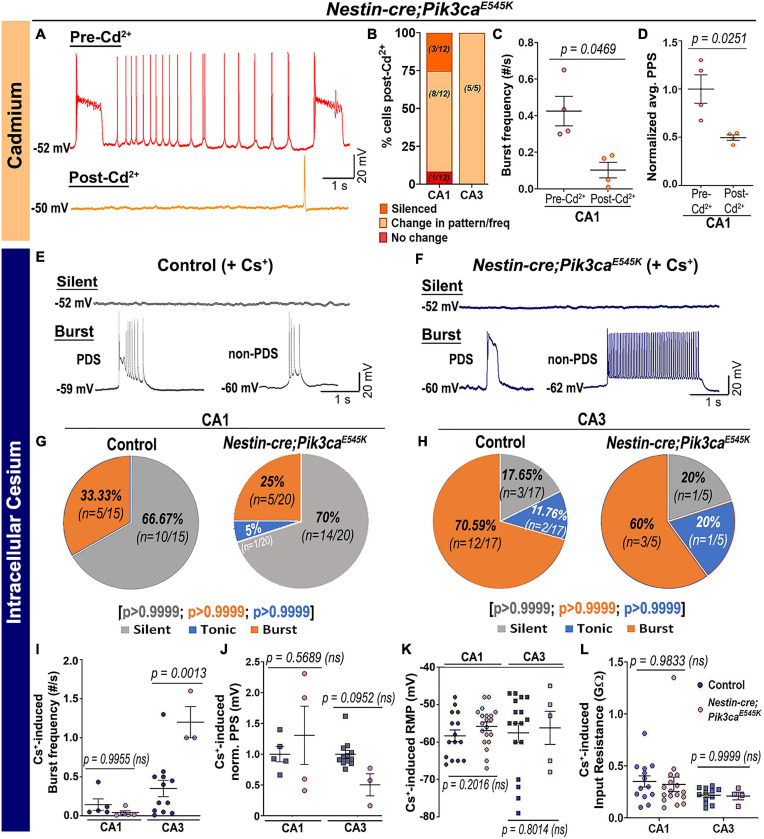
Pik3ca-related burst characteristics are dependent on cell-intrinsic calcium and potassium channel regulation. **(A–D)** Blocking calcium channels by extracellular cadmium (Cd^2+^) significantly altered the intrinsic bursting pattern of mutant neurons including burst frequency (*t* = 3.267, df = 3) and PPS (*t* = 4.172, df = 3). **(E–H)** Representative traces and respective pie charts show types of cellular activity in control and mutant CA1 and CA3 neurons after administering cesium intracellularly. In response to cesium, tonic firing cells were significantly reduced and original differences in CA1 and CA3 cell proportions between control and mutant were normalized. **(I)** Cs^+^-induced burst frequency was significantly higher in mutant CA3 than those in respective controls (*F* = 26.31, df = 21); unlike normal spontaneous state, no such difference was observed in CA1. **(J–L)** Intracellular cesium lowered the spontaneously enhanced mutant PPS in CA1 to control levels (*F* = 4.003, df = 20), while having no effect on RMP (CA1: *t* = 1.308, df = 27.32; CA3: *t* = 0.2616, df = 6.816) or input resistance (*F* = 2.936, df = 42) in both control and mutant neurons. Data is represented as pie charts, % bar graphs and mean ± SEM scatter plots; differences were considered significant at *p* < 0.05; ns, not significant. Scale bars: 1 s, 20 mV **(A,E,F)**.

In contrast, inhibition of calcium (Ca^2+^)-dependent inward current by extracellular cadmium (Cd^2+^) attenuated the paroxysmal bursts and reduced burst frequency and PPS (Figures 5A–D), indicating a calcium channel-dependent mechanism underlying the Pik3ca-dependent seizure activity. Intracellular cesium blocked potassium channels and related currents, altering the intrinsic firing pattern in both control and mutant hippocampal slices. Specifically, compared to regular baseline recordings, intracellular cesium considerably reduced the proportion of tonic-firing cells in both CA1 and CA3 (Figures 5E–H, compare to Figures 2D,E). Unlike the spontaneous recordings, intracellular cesium prompted the burst frequency in mutant CA1 to normalize and in mutant CA3 to significantly rise, relative to respective controls (Figure 5I, compare to Figure 2G). Intracellular cesium also normalized the mutant PPS to control levels (Figure 5J, compare to Figure 2M). No overt effect on RMP or input resistance was seen in cesium-treated control and mutant cells (Figures 5K,L). We conclude that Pik3ca-related epileptiform activity is primarily caused by a complex set of altered non-synaptic cell-intrinsic properties.

### Acute Treatment of Neuronal Hyperactivation With PI3K Drugs

**FIGURE 6:**
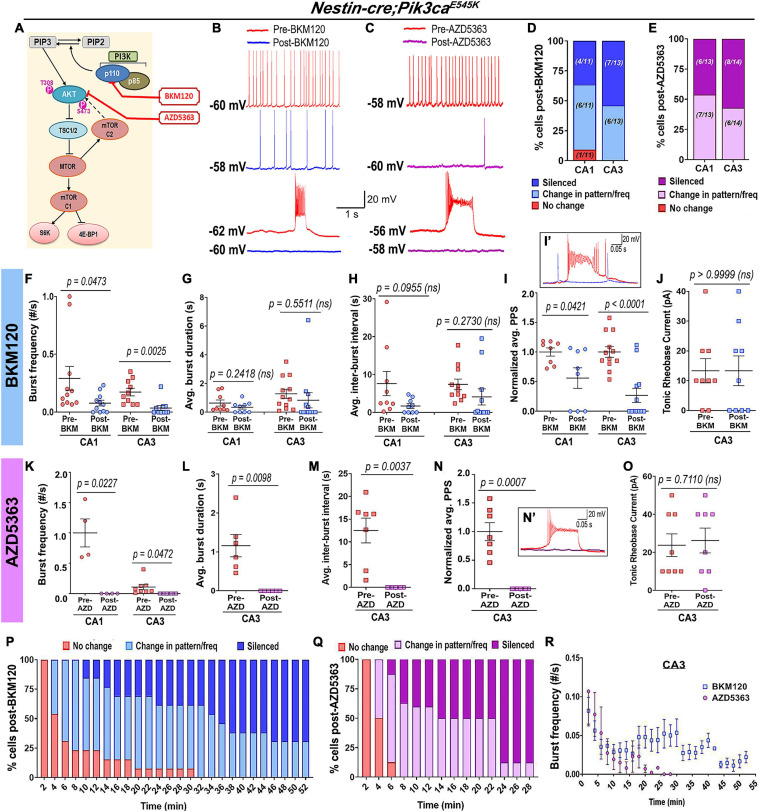
Acute inhibition of Pik3ca or AKT suppresses hyperactivity in mutant neurons. **(A)** Schematic of simplified PI3K-AKT-MTOR pathway, marking targets of action for BKM120, AZD5363, and RAD001. Representative traces demonstrate differential action of BKM120 **(B)** and AZD5363 **(C)** on *Nestin-cre;Pik3ca^*E545K*^* hippocampal neurons, ranging from being unaffected to change in frequency/pattern to silencing, as quantitated, respectively, in panels **(D,E)**. **(F–J)** Acute BKM120 treatment in the mutant neurons significantly reduced burst frequency (*F* = 8.937, df = 38) and PPS (*F* = 24.39, df = 36) in both CA1 and CA3; but did not significantly affect the average burst duration (*t* = 1.073, df = 4), inter-burst interval (*t* = 0.4023, df = 4) or rheobase current (*t* = 1.000, df = 4). **(K–O)** Acute AKT inhibition in mutant slices by AZD5363 significantly reduced burst frequency (*F* = 43.50, df = 20), PPS (*t* = 6.386, df = 6), average burst duration (*t* = 4.052, df = 5) and inter-burst interval (*t* = 4.597, df = 6), largely by suppressing bursts or silencing the cells. AZD5363 had no effect on the mutant rheobase current (*t* = 0.3859, df = 7). **(I’,N’)** Insets show activity traces of mutant bursting cell before (red) and after BKM120 (blue) or AZD5363 (pink) administration. Plots marked percentage of mutant CA3 “bursting” cells undergoing changes as a function of time, in response to acute administration of BKM120 **(P)** and AZD5363 **(Q)**. **(R)** Mutant plateau frequency significantly dropped as a function of time, where 0 min marked the time of administration of BKM120/AZD5363 onto the brain slice. Data is represented as % bar graphs and mean ± SEM scatter plots; differences were considered significant at *p* < 0.05; ns, not significant. Each pair of pre-drug (spontaneous recording) and post-drug values (from final 5 min recording) were obtained from the same cell **(F–O)**. Scale bars: 1 s, 20 mV **(B,C)**; 0.05 s, 20 mV **(I’,N’)**.

The acute *in vivo* suppression of induced seizures in *Nestin-cre;Pik3ca^*E545K*^* mice by BKM120 (Roy et al., 2015) prompted us to dissect the downstream pathway dynamics using inhibitors at the cellular level, in order to coarsely determine their mechanistic roles and identify new therapeutic targets to treat intractable epilepsy. Acute extracellular administration of BKM120 or the pan-AKT inhibitor AZD5363 (Davies et al., 2012; Figures 6A–F,I,I’,K,N,N’) resulted in a large-scale and significant alteration of firing pattern, frequencies and a relative reduction of PPS, sometimes leading to gradual silencing of the recorded neurons. In contrast to BKM120, acute AKT downregulation by AZD5363 significantly reduced the mutant burst duration and inter-burst interval (Figures 6G,H,L,M). Neither drug had any significant effect on neuronal RMP (pre-BKM120_CA1_: −58.7 ± 1.89 mV, post-BKM120_CA1_: −56.1 ± 1.38 mV; pre-BKM120_CA3_: −59.5 ± 2.07 mV, post-BKM120_CA3_: −58.58 ± 1.70 mV; pre-AZD5363_CA1_: -55.25 ± 2.63 mV, post-AZD5363_CA1_: −52.5 ± 2.18 mV; pre-AZD5363_CA3_: −56.25 ± 2.79 mV, post-AZD5363_CA3_: −54.75 ± 3.95 mV) or tonic rheobase current (Figures 6J,O). Further, AZD5363 altered the physiology of all mutant CA3 neurons much faster (∼8 min) than BKM120 (∼32 min); however, both drugs changed the intracellular activity of ∼50% cells within 4 min. AZD5363 also blocked spontaneous bursts in mutant neurons faster than BKM120 (Figures 6P–R). Thus, acute regulation of either PI3K or AKT activity directly suppressed *Pik3ca*^*E545K*^-driven epileptiform neuronal activity.

**FIGURE 7:**
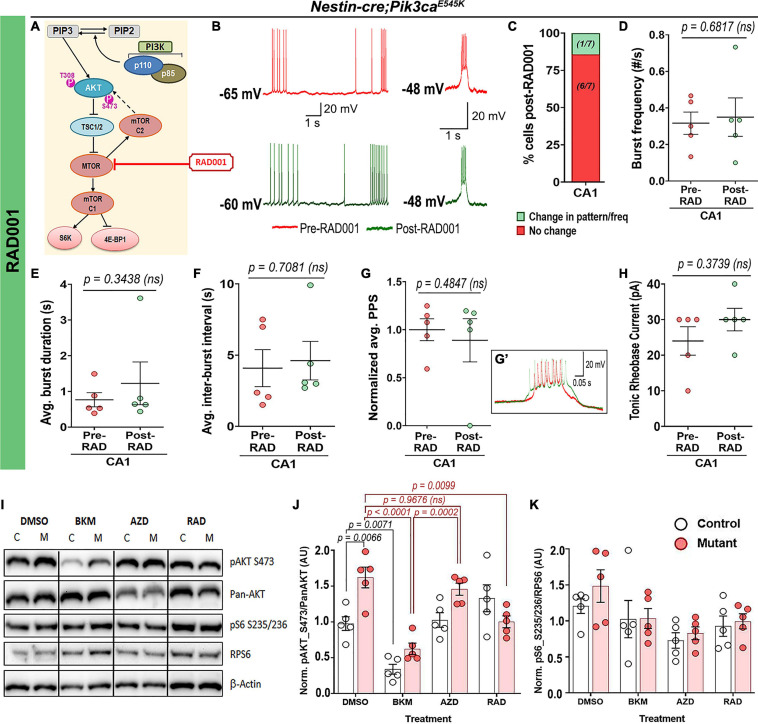
Acute treatment with RAD001 has no overt effect on mutant neuronal physiological properties. **(A)** Schematic of simplified PI3K-AKT-MTOR pathway, marking target of action for RAD001. Representative traces demonstrate action of RAD001 **(B)** on *Nestin-cre;Pik3ca^*E545K*^* hippocampal neurons, ranging from being unaffected to change in frequency/pattern to silencing, as quantitated, respectively, in panel **(C)**. **(D–H)** Acute RAD001 treatment had no overt effect on the mutant burst frequency (*t* = 0.4414, df = 4), average burst duration (*t* = 1.073, df = 4), inter-burst interval (*t* = 0.4023, df = 4), rheobase current (*t* = 1.000, df = 4) or PPS (*t* = 0.7692, df = 4). **(I)** Representative Western blots bands of pAKT_S473, pan-AKT, pS6-S235/236 and RPS6 (total S6) are demonstrated across different treatment (DMSO, BKM120, AZD5363, and RAD001). **(J,K)** Quantifications of normalized ratios of phosphoproteins over total proteins showed significant reduction of pAKT_S473 in response to acute BKM120 and RAD001 treatment but not post-AZD5363 (*F* = 22.98, df = 32); pS6_S235/236 in mutant had a decreasing trend in response to all pathway drugs (*F* = 4.485, df = 32). Data is represented as mean ± SEM scatter plots and bar graphs; differences were considered significant at *p* < 0.05; ns, not significant. Each pair of pre-RAD (spontaneous recording) and post-RAD values (from final 5 min recording) were obtained from the same cell **(D–H)**. Scale bars: 1 s, 20 mV **(B)**; 0.05 s, 20 mV **(G’)**.

In contrast, acute administration of the MTOR inhibitor RAD001 (everolimus) (Krueger et al., 2010; Cho, 2011) showed no overt effect on the mutant RMP (pre-RAD001_CA1_: −59 ± 3.55 mV, post-RAD001_CA1_: −55.6 ± 2.04 mV), tonic or burst firing patterns, frequencies, average PPS, burst duration, inter-burst interval and rheobase current (Figures 7A–H). This was despite western blot confirmation that acute treatment of forebrain slices with RAD001 modulated direct downstream targets of MTOR (Figures 7I–K). In summary, our study demonstrates that neuronal hyperactivity caused by *Pik3ca* gain-of-function mutation is based on multiple intrinsic electrophysiological characteristics that are acutely modifiable by downregulating PI3K and AKT activity, but not MTOR.

## Discussion

Activating mutations in the PI3K-AKT-MTOR pathway commonly cause cortical malformations and pediatric intractable epilepsy (Dobyns and Mirzaa, 2019; Kim and Lee, 2019); yet the underlying mechanisms remain largely undefined. MTOR overactivation and chronic post-transcriptional mechanisms have been widely emphasized as central to these phenotypes (Cho, 2011; Berdichevsky et al., 2013; Meng et al., 2013; Cardamone et al., 2014; Kim and Lee, 2019). However, we have shown that dysmorphology caused by activating *Pik3ca* mutations is distinct from that resulting from MTOR overactivation (Roy et al., 2015). Here we wanted to address if epilepsy mechanisms are also distinct. Using our *Nestin-cre;Pik3ca^*E545K*^* model, we establish that Pik3ca-dependent neuronal hyperactivation in hippocampal pyramidal neurons is primarily driven by intrinsic neuronal mechanisms, independent of synaptic inputs. Acute attenuation of seizure activity by inhibiting AKT but not MTOR also reveals a previously unknown role of PI3K signaling in neuronal homeostasis. The very short time of action implicates post-translational versus -transcriptional mechanisms.

Epileptiform activity is typically marked by increased tonic depolarizations and/or quasiperiodic bursts, mediated by intrinsic membrane properties, ephaptic or synaptic/circuit-level interactions (Dudek et al., 1998; McCormick and Contreras, 2001). We confirmed enhanced interictal spike frequency and diverse, synchronized epileptiform spike patterns in the mutant brains *in vivo*, predominantly in the hippocampus. This is consistent with studies of *PIK3CA* variants in brain tumors and mouse models with patient-related *Pik3r2* mutations, which are less common causes of intractable epilepsy (Shi et al., 2020; Yu et al., 2020). Our *ex vivo* analyses using acute brain slices revealed intracellular correlates of epileptiform activity and enhanced firing frequencies in the mutant hippocampal pyramidal neurons. Relative to controls, *Pik3ca*^*E545K*^ neurons exhibited significantly higher proportions of bursting cells and higher heterogeneity in burst patterns, including burst clusters and PDSs. A prior study showed that human neurons from surgically resected neocortical samples of pediatric patients with FCD exhibited remarkably similar burst characteristics (Marcuccilli et al., 2010). These samples were not genotyped; however, since most cases of FCD and epilepsy result from variants in PI3K pathway genes (Mirzaa et al., 2018), such cross-species phenotypic correlation is remarkable and emphasizes the clinical relevance of our mouse model.

The PI3K-AKT-MTOR pathway is known to influence synaptogenesis and neuronal plasticity (Sanchez-Alegria et al., 2018). For example, neocortical samples from patient and mouse models with *TSC* mutations exhibit synaptic input-mediated epileptogenic hyperexcitability (Wang et al., 2007). However, blocking glutamatergic (NMDA and non-NMDA) or GABAergic synaptic inputs in our mutant brain slices showed no significant effect on the neuronal activity. Thus, our data demonstrate that altered synaptic interactions are not primary mediators of *Pik3ca*^*E545K*^-driven acute hippocampal neuronal hyperactivation. We acknowledge, however, that altered neuronal circuitry are likely contributors to chronic mechanisms driving Pik3ca-mediated epilepsy. Regardless, for the first time, our data indicates that acute seizure mechanisms are distinct and dependent on the location of the mutant protein within the PI3K-AKT-MTOR pathway. This conclusion is further supported by a recent study of a mouse model of MTOR activation, where neuronal hyperexcitation was found be driven by non-cell autonomous mechanisms and not associated with altered intrinsic properties (Koh et al., 2021). Significant reduction of burst-generating cells, burst frequency and PPS by blocking voltage-dependent calcium or potassium channels indicate that *Pik3ca*^*E545K*^-driven hyperactivity is predominantly mediated by multiple altered cell-intrinsic properties. Yet, some cell-intrinsic properties, such as burst frequencies, PPS and time constants, are distinctly different between mutant CA1 and CA3 neurons. Since all neurons do not react uniformly to either pathway overactivation or Cd^2+^/Cs^+^-dependent channel inhibition, current anti-seizure drugs that typically target single ion channels cannot address the heterogeneous dysregulation, thus potentially explaining epileptic intractability in this patient population. It is intriguing to note that intercellular signaling heterogeneity involving the PI3K-AKT-MTOR pathway was also reported by a recent quantitative study that highlighted its potential relevance to human disorders, such as insulin resistance (Norris et al., 2021).

PI3K-AKT-MTOR inhibitors developed to treat cancer potentially represent novel anti-seizure therapeutics (Maira et al., 2012; Cardamone et al., 2014; Lindhurst et al., 2015; Venot et al., 2018; Zou et al., 2020; Forde et al., 2021). Rapamycin and its analogs, that are currently in clinical use to treat epilepsy in TSC patients, curbed epileptiform activity in mouse models harboring *TSC1/2*, *RHEB, MTOR*, or *PTEN* mutations when administered long-term (>7-day) (Curatolo and Moavero, 2013; Lasarge and Danzer, 2014; Crino, 2016; Kim and Lee, 2019; Stafstrom, 2019; Hsieh et al., 2020). In contrast, we show acute inhibition of mutant neuronal hyperactivity by BKM120 at cellular level, confirming our previous *in vivo* findings (Roy et al., 2015). We also demonstrate that acute AKT inhibition with AZD5363 has similar effects. This first preclinical study using AZD5363 shows that acute Pik3ca-driven seizure mechanism is predominantly AKT-dependent, limiting roles for other PI3K targets. Intriguingly, rapamycin analog RAD001 had minimal acute effect in our model, yet western analyses demonstrated pathway inhibition with RAD001 was similar to that seen with the other pathway drugs we assessed. While elevated MTOR activity is known to be epileptogenic (Crino, 2016; Kim and Lee, 2019; Koh et al., 2021), our data suggest that alternate PI3K-AKT targets acutely regulate neuronal hyperactivation in PIK3CA-driven epilepsy.

Our study establishes an important foundation to determine active pathway-driven cellular mechanisms in Pik3ca-related epilepsy. The mechanisms are clearly complex. Additional studies will define differential involvement of specific subtypes of Ca^2+^ and K^+^ channels in each type of neuron. However, as a baseline, we have identified specific *ex vivo* parameters to assess the plethora of additional available PI3K pathway inhibitors, to facilitate the development of new molecularly rational therapeutic interventions for intractable epilepsy.

## Materials and Methods

### Mice

The following mouse lines were used: *Nestin-cre* (Jackson Labs, Bar Harbor, ME, United States; Stock #003771, RRID: IMSR_JAX:003771), conditional *Pik3ca*^*E545K*^ knock-in (Robinson et al., 2012). *Nestin-cre* and *Pik3ca^*E545K/*+^* mouse lines were maintained in C57BL/6 and FVB backgrounds, respectively; the progeny mice are of mixed background. The mutant activating *Pik3ca*^*E545K*^ allele is heterozygous, as in human patients. We have designated *Nestin-cre;Pik3ca^*E545K*/+^* conditional mutant mice as “*Pik3ca* mutants” or “mutants” throughout the manuscript.

All mice were housed in Optimice cages with aspen bedding at the Seattle Children’s Research Institute’s specific pathogen-free (SPF) vivarium facility (light “ON”: 6 a.m.–8 p.m.). Noon of the day of vaginal plug was designated as embryonic day 0.5 (E0.5). The day of birth was designated as postnatal day 0 (P0). Genotyping by PCR was done using separate sets of primers for the *Cre* coding region and the *Pik3ca*^*E545K*^ allele, as previously described (Roy et al., 2015). All mouse procedures were approved and conducted in accordance with the guidelines laid down by the Institutional Animal Care and Use Committees (IACUC) of Seattle Children’s Research Institute, Seattle, WA, United States. ARRIVE guidelines have been followed for reporting work involving animal research.

### *In vivo* Electrophysiology

Five control and six *Nestin-cre;Pik3ca^*E545K*^* mutant mice (age: ∼P70) were used for *in vivo* regular and depth-electrode electrophysiology experiments. We saw no sex-dependent data correlation (Roy et al., 2015).

#### Electrode Implantation Surgery

Mice underwent survival surgery to implant ECoG, EMG, and hippocampal depth electrodes under isoflurane anesthesia using similar procedures as reported in our prior work (Roy et al., 2015; Bolea et al., 2019). The ECoG electrodes consisted of silver wire (diameter: 130 μm bare; 180 μm coated) attached to micro-screws. The ECoG electrodes were implanted bilaterally through small cranial burr holes above the somatosensory cortices. A similar reference electrode was placed above the cerebellum following the same procedure. The depth electrodes were made of 2 fine twisted tungsten wires (30 μm nylon coated, California fine wire) and implanted in the hippocampal CA1 region (coordinates: −2.0 mm anteroposterior, 1.5 mm mediolateral, 1.9 mm dorsoventral in reference to bregma) to record LFP. All electrodes were connected to interface connector and fixed to the skull with dental cement (Lang Dental Manufacturing Co., Inc., Wheeling, IL, United States). Mice were allowed to recover from surgery for 2–3 days.

#### Video, Electroencephalography, and Local Field Potential Recording

Simultaneous video-EEG-LFP recordings were collected from conscious mice on PowerLab 8/35 and 16/30 data acquisition units, using LabChart 7.3.3 software (AD Instruments, Colorado Spring, CO, United States). ∼6 h of baseline ECoG tracings were visually reviewed for the presence of spontaneous epileptiform events, as previously studied (Kalume, 2013; Liautard et al., 2013; Roy et al., 2015). All bioelectrical signals were acquired at a 20 KHz sampling rate. The ECoG signals were processed with a 1–70 Hz bandpass filter and the LFP signal with a 5 Hz high-pass filter. Interictal spikes were identified as transient, clearly distinguished from background activity, with pointed peak and slow wave. Myoclonic seizures were identified as shock-like muscular jerks on video, associated with a spike or polyspike-wave complex on EEG. Power spectral analysis and visual inspection of the data were conducted to characterize the EEG activity in different frequency bands and identify epileptiform events on the ECoG and local CA1 field recordings. The different frequency bands used in the study (Delta: 0.5–5 Hz, Theta: 5–10 Hz, Gamma: 20–30 Hz) are based on our previously published parameters (Hughes, 2008; Kalume et al., 2015).

### *Ex vivo* Electrophysiology

#### Slice Preparation

Postnatal (P16–20) pups were anesthetized briefly in a closed chamber by administering isoflurane (5% flow rate) or CO_2_ (constant flow rate: 10–30% of chamber vol/min); then perfused transcardially with ice-cold low Na^+^-buffer (“slicing solution,” which included the following: 252 mM sucrose, 2 mM KCl, 2 mM MgCl_2_, 2.6 mM CaCl_2_, 1.2 mM NaH_2_PO_4_, 26 mM NaHCO_3_, and 15 mM glucose, with the pH adjusted to 7.4 and the osmolarity to 310 ± 5 mOsm). Brain was dissected out by separating the head and cutting along the skull sutures using fine scissors and forceps. The forebrain was isolated in ice-cold, oxygenated (95% O_2_, 5% CO_2_) slicing solution. A slanted (∼15° from vertical) agar block was secured on a specimen tray as a support for the brain during slicing. The isolated forebrain was oriented for horizontal slicing, glued to the specimen tray with cyanoacrylate and placed in the vibratome for slicing. Once the hippocampus became visible, acute horizontal slices of ∼250 μm thickness were collected and immediately incubated in the same slicing solution, maintained in a warm bath (28 ± 0.5°C) for recovery. After 30 min, they were transferred into regular artificial cerebrospinal fluid (aCSF), composed of the same components as slicing solution except for the replacement of sucrose with 126 mM NaCl. Slices were then kept at room temperature, continuously superfused with oxygenated aCSF, until recording. Given that *Pik3ca*^*E545K*^ mutant is megalencephalic, a greater number of hippocampal/forebrain sections were obtained. However, care was taken so that the dorso-ventral plane(s) used for recording was always comparable between control and mutant slices. No randomization was used. Tissue collection was not performed blind since the mice were subjected to genotyping and drug administration.

#### Recording

Slices were transferred into a recording chamber, continuously superfused with oxygenated aCSF, for *ex vivo* whole-cell recordings. The pClamp software suite (Molecular Devices; RRID: SCR_011323) was used for data acquisition and analysis. Signals were amplified (MultiClamp 700A, Axon Instruments, Molecular Devices, United States), digitized (D1322A, Axon Instruments, Molecular Devices, United States), and stored in a computer for post-hoc analysis. Horizontal hemi-forebrain slices with prominent hippocampus were used for intracellular whole-cell visual patch-clamp experiments. Slices transferred to the recording chamber were maintained at 30–34°C, constantly superfused with oxygenated aCSF Borosilicate glass capillaries/pipettes for patch-clamp recording had electrode resistance (R_e_) optimally kept around 5–8 MΩ, after being filled with internal solution containing the following: potassium gluconate (∼132 mM), KCl (5 mM), HEPES (10 mM), EGTA (5 mM), CaCl_2_.H_2_O (0.5 mM), MgCl_2_ (2 mM), disodium phosphocreatine (5 mM), disodium-ATP (4 mM), trisodium-GTP (0.5 mM), EGTA (5 mM). Cells were visualized under brightfield optics using the 40X water-immersion objective of an upright microscope (Olympus, BX51WI). The patch electrode was advanced toward the target cells by a micromanipulator (MP-225, Sutter Instrument Company, United States) and 1 GΩ seal was established, typically by a small negative pressure, with the membrane ruptured by gentle suction and/or zap pulses. Whole-cell patch-clamp recording was performed from cell bodies of pyramidal neurons of hippocampal CA1 and CA3, respectively. Following intracellular recording protocols were also used:

(i) Spontaneous cell-attached gapfree recordings in current (I)-clamp: cells were typically tested at resting conditions (without current injection) unless noted otherwise.
(ii) Evoked I-clamp steps protocol: current steps start at −50 pA, incremental 10 pA, duration; 300 ms; 15 steps (−50 to +90 pA) were recorded across experiments.

For the *ex vivo* patch-clamp recording of CA1 and CA3 pyramidal neurons, we used on average 1–2 slices per mouse per genotype and recorded >1 cell per slice. We were meticulous in using similar planar positions of brain slices for our experiments. It is possible there were unconscious sampling biases based on the neuronal position within the CA1 or CA3 pyramidal layer per brain slice.

### Acute Chemical Assays

#### Channel Blockers

Chemicals inhibiting specific ion channels were introduced in the bath/recording buffer to compare different physiological components possibly contributing to the neuronal hyperactivity. To block all fast-synaptic excitatory transmission, 3-[(±)2-carboxypiperazin-4yl] propyl-1-phosphate (CPP, NMDA receptor antagonist, 20 μM; Tocris Bioscience, United Kingdom) and 6-cyano-7-nitroquinoxaline-2,3-dione [CNQX, AMPA/kainate (non-NMDA) receptor antagonist, 20 μM, diluted in DMSO; Alomone Labs, Israel] were introduced in the bath. SR95531 hydrobromide or Gabazine (GABA_A_ receptor antagonist, 10 μM, Tocris Bioscience, United Kingdom) was used to block inhibitory synaptic transmission. Cd^2+^ (CdCl_2_, 100 μM; Sigma-Aldrich, United States) was used to depress synaptic transmission and block inward calcium-selective current, isolating the outward K^+^ current. To block voltage-dependent K^+^ channels, intracellular administration of cesium was done by replacing potassium gluconate with cesium gluconate in the internal solution while maintaining the same osmolarity.

#### Phosphoinositide-3-Kinase Pathway Drugs

Phosphoinositide-3-kinase pathway inhibitors dissolved in 100% DMSO were added to the circulating recording buffer, thus getting acutely administered in the entire brain slice that is being recorded. For protein analyses, these were added at identical concentration to the brain slices incubated in oxygenated aCSF for ∼1 h. The following inhibitors were used *ex vivo*: pan-PI3K inhibitor BKM120 (Buparlisib; Novartis, Switzerland; working concentration: 3.5 μM); pan-AKT inhibitor AZD5363 (Capivasertib; Selleckchem, United States; working concentration: 0.5 μM); MTOR inhibitor RAD001 (Everolimus, Chem Express Cat# 159351-69-6; working concentration: 0.52 μM). To determine the AZD5363 dose needed to inhibit the AKT isoforms and phosphorylation of AKT in the acute brain slices of our mouse model, we followed the datasheet from https://www.selleckchem.com/products/azd5363.html. A survey of literature demonstrates multiple electrophysiological parameters in wild-type and various mutant (including *TSC2+/-*) mouse brain slice assays with acute drug exposures from 10 nM through 1 μM. The drug concentrations we chose for our study are well within this range (Tang et al., 2002; Ehninger et al., 2008; Olde Engberink et al., 2017; Artinian et al., 2019).

### Western Blotting

Horizontal forebrain slices from five control and five mutant mice were obtained and recovered in oxygenated aCSF as detailed above. Slices showing nice hippocampal morphology were selected, treated for 1 h with DMSO, BKM120, AZD5363, and RAD001, respectively, in the same concentrations used for recording. The treated slices were then flash-frozen in liquid nitrogen and stored at −80°C for post-processing. These brain slices were lysed in NP-40 lysis buffer [150 mM NaCl, 50 mM Tris (pH 7.4), 1% NP-40, 10 mM NaF, 2 mM sodium orthovanadate + protease/phosphatase inhibitor cocktails (Sigma, United States)]. Samples were normalized to equal protein concentrations (0.333 mg/ml) using Pierce BCA protein assay (Thermo Fisher Scientific, United States). Standards were made through serial dilutions of 10 mg/ml BSA. Samples were diluted into a final concentration of 1x Laemmli sample buffer and boiled at 95°C for 10 mins. 7.5% 10-well gels were prepared, and samples were run at 120 V for 1.5 h. Gels were transferred onto polyvinylidene difluoride (Millipore) membranes and run either overnight at 25 V or for 2 h at 60 V. Membranes were blocked in 4% milk in TBST (0.05 M Tris, 0.15 M NaCl, pH 7.2, 0.1% (v/v) Tween20) for 1 h at room temperature, and primary antibodies were applied overnight at 4°C in blocking medium. Primary antibodies for western blots were diluted as follows: rabbit anti-Phospho-AKT Ser473 (D9E) (Cell Signaling Technology, United States; RRID: AB_2797780; 1:2,000), mouse anti-Pan-AKT (40D4) (Cell Signaling Technology, United States; Cat# 2920; 1:2,000), rabbit anti-Phospho-S6 Ribosomal Protein Ser235/236 (Cell Signaling Technology, United States; RRID: AB_2721245, 1:2,000), mouse anti-S6 Ribosomal Protein (54D2) (Cell Signaling Technology, United States; RRID: AB_2238583, 1:1,000), rabbit anti-beta(β)-Actin (GeneTex, United States; RRID: AB_1949572, 1:10,000). After washing and probing, respectively, with goat anti-rabbit (RRID: AB_2313567) and anti-mouse (RRID: AB_10015289) horseradish peroxidase (HRP)-conjugated secondary antibodies (1:10,000; Jackson ImmunoResearch Labs, United States), blots were imaged using Femto chemiluminescent detection reagents (Thermo Fisher Scientific, United States; Cat# 34095) in a FluorChem R western blot imaging system (ProteinSimple, Bio-Techne, United States). 8-bit images were used as a representative western blot. 16-bit images were used to quantify the intensity of each band using ImageJ v1.53. Regions of interest were drawn in each sample’s lane. After quantifying the lane in a histogram, the peak representing the band of interest was isolated and the area of the region was measured as the band’s quantification.

### Quantitative and Statistical Analyses

Number of mice used was consistent with previous experiments completed and published by us and other investigators. In whole-cell patch-clamp recording, PPS was calculated by subtracting the baseline potential from the highest plateau/burst potential in a paroxysmal depolarizing event. Burst duration and inter-burst interval were measured as shown in Figure 2 and averaged across multiple bursts per cell. Burst frequency was calculated as number of burst episodes per time interval. Input resistance was measured from evoked current-clamp recordings by dividing the voltage difference (measured at −10 to −30 pA I-steps) by the current interval of 20 pA. The decay membrane time constant was obtained by recording the membrane response to 20 pA hyperpolarizing current pulses (300 ms duration, 1 Hz) and fitting the response to a single exponential curve. We chose 20 pA of hyperpolarizing current because such a current intensity did not produce sag. Evoked spike frequencies and rheobase current were calculated exclusively from tonic-firing cells. Burst threshold current was calculated from burst-generating cells as the first current step that induced burst. Whole-cell electrophysiological analyses were performed using Clampfit 10.7 and 11 (pClamp, Molecular Devices, United States). For Western Blots, each band intensity was recalculated relative to its respective b-actin band intensity, and then normalized across average intensity per protein lane. Normalized ratios of phosphoproteins over total proteins were thereafter obtained.

Statistical significance was assessed using 2-tailed unpaired *t*-tests with Welch’s correction (EEG power analyses, RMP, burst duration, inter-burst interval, Cd^2+^ data), 2-tailed paired *t*-tests (rheobase current, drug-treated analyses) and ANOVA followed by Tukey post-tests (EEG interictal spike frequency, whole-cell tonic spike and burst frequencies, cell proportions, PPS, evoked spike frequency, time constant; Western blots). Normal distribution was assumed for data analyses, as required. For EEG-ECoG-LFP experiments, statistical data analysis was performed in Labchart 8.2 software (AD Instruments) and Igor Pro 6.37 (WaveMetrics Inc., United States); final graphs were made in GraphPad Prism v7.0 (GraphPad Software Inc., San Diego, CA, United States). For the remaining data, statistical analyses and graph plotting were done using GraphPad Prism v7.0 and Microsoft Excel. Differences were considered significant at *p* < 0.05.

## Data Availability Statement

The original contributions presented in the study are included in the article/supplementary material, further inquiries can be directed to the corresponding authors.

## Ethics Statement

The animal study was reviewed and approved by Institutional Animal Care and Use Committees (IACUC) of Seattle Children’s Research Institute, Seattle, WA, United States.

## Author Contributions

AR, FK, J-MR, and KJM contributed to the study conception and design and to the overall data interpretation. AR, VH, AMB, DTW, and FK contributed to the data collection. AR and FK contributed to *ex vivo* and *in vivo* data analyses, respectively. DTW and SEPS contributed to western blot data analysis and interpretation. AR, SEPS, FK, and KJM provided the funding resources. AR wrote the first draft of the manuscript and others commented on previous versions of the manuscript. All authors read and approved the final manuscript.

## Conflict of Interest

The authors declare that the research was conducted in the absence of any commercial or financial relationships that could be construed as a potential conflict of interest.

## Publisher’s Note

All claims expressed in this article are solely those of the authors and do not necessarily represent those of their affiliated organizations, or those of the publisher, the editors and the reviewers. Any product that may be evaluated in this article, or claim that may be made by its manufacturer, is not guaranteed or endorsed by the publisher.
